# Supplementary figures and images for: The Cost and the Value of Stroke Care in Greece: Results from the SUN4P Study
Source: Healthcare (Basel). 2023 Sep 14;11(18):2545. doi: 10.3390/healthcare11182545 (PMC10530928; doi:10.3390/healthcare11182545)

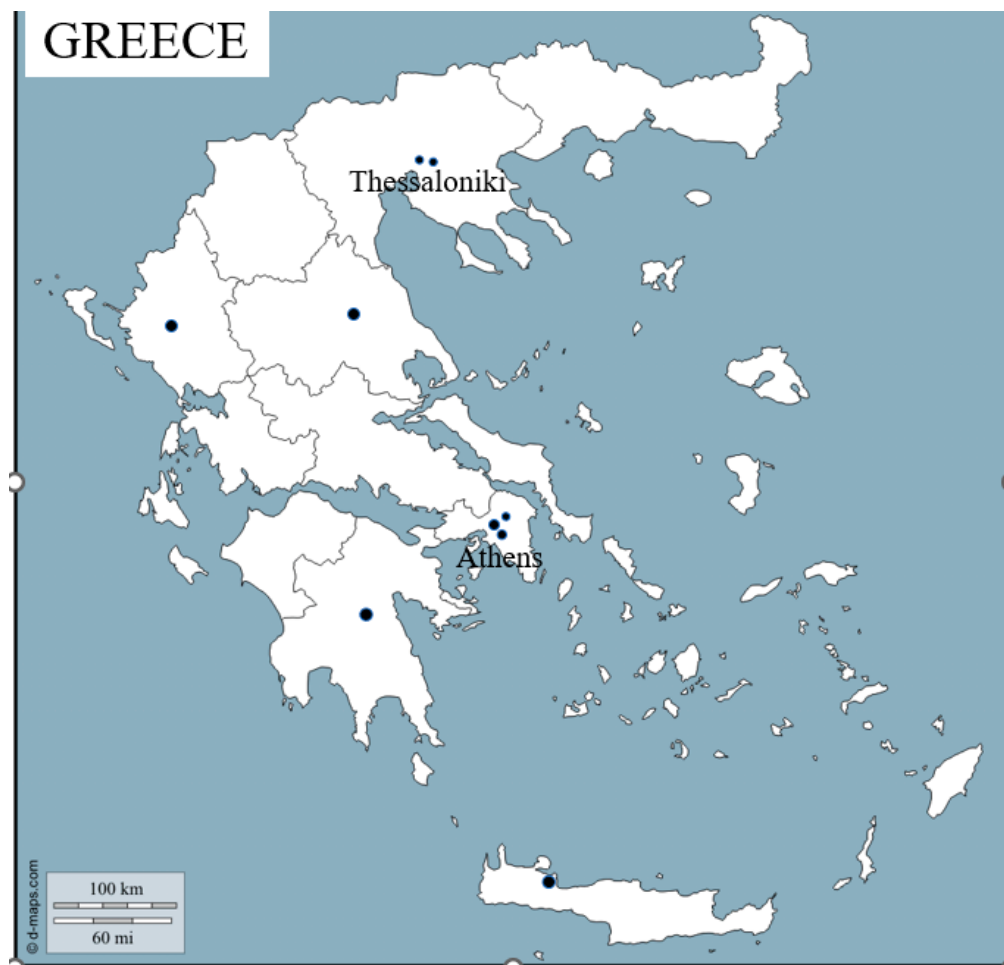

**Figure S1.** Sites of participating study centers.

Supplement: Supplementary file 1 [file healthcare-11-02545-s001.zip › healthcare-2552569-supplementary.pdf]
